# Surfactant Protein D modulates allergen particle uptake and inflammatory response in a human epithelial airway model

**DOI:** 10.1186/1465-9921-13-8

**Published:** 2012-02-01

**Authors:** Carsten Schleh, Barbara M Rothen-Rutishauser, Fabian Blank, Hans D Lauenstein, Matthias Nassimi, Norbert Krug, Armin Braun, Veit J Erpenbeck, Peter Gehr, Jens M Hohlfeld

**Affiliations:** 1 Fraunhofer Institute of Toxicology and Experimental Medicine, Nikolai-Fuch-Str. 1, 30635 Hannover, Germany; 2 MedicalSchoolHannover, Carl-Neuberg-Str. 1, 30625 Hannover, Germany; 3 Respiratory Medicine, Berne University Hospital, Murtenstrasse 50, Postfach 44, 3010 Bern, Switzerland; 4 Adolphe Merkle Institute, University of Fribourg, Rte de l'Ancienne Papeterie CP 209, 1723 Marly 1, Switzerland; 5 Technical University Carolo-Wilhelmina Braunschweig, 38092 Braunschweig Germany

**Keywords:** Allergen Particle, Subpollen Particles, SPP, Surfactant Protein D, SP-D, Cytokines

## Abstract

**Background:**

Allergen-containing subpollen particles (SPP) are released from whole plant pollen upon contact with water or even high humidity. Because of their size SPP can preferentially reach the lower airways where they come into contact with surfactant protein (SP)-D. The aim of the present study was to investigate the influence of SP-D in a complex three-dimensional human epithelial airway model, which simulates the most important barrier functions of the epithelial airway. The uptake of SPP as well as the secretion of pro-inflammatory cytokines was investigated.

**Methods:**

SPP were isolated from timothy grass and subsequently fluorescently labeled. A human epithelial airway model was built by using human Type II-pneumocyte like cells (A549 cells), human monocyte derived macrophages as well as human monocyte derived dendritic cells. The epithelial cell model was incubated with SPP in the presence and absence of surfactant protein D. Particle uptake was evaluated by confocal microscopy and advanced computer-controlled analysis. Finally, human primary CD4^+ ^T-Cells were added to the epithelial airway model and soluble mediators were measured by enzyme linked immunosorbent assay or bead array.

**Results:**

SPP were taken up by epithelial cells, macrophages, and dendritic cells. This uptake coincided with secretion of pro-inflammatory cytokines and chemokines. SP-D modulated the uptake of SPP in a cell type specific way (e.g. increased number of macrophages and epithelial cells, which participated in allergen particle uptake) and led to a decreased secretion of pro-inflammatory cytokines.

**Conclusion:**

These results display a possible mechanism of how SP-D can modulate the inflammatory response to inhaled allergen.

## Background

Over the past 30 years asthma prevalence has markedly increased. A high percentage of asthmatics is sensitized against pollen [1]. Whole pollen are large in size (up to 50 μm) so that they preferentially deposit in the upper airways with a very low fraction reaching the deeper lung upon inhalation. However, allergen loaded subpollen particles (SPP, also named pollen starch granules (PSG)) are released from whole pollen upon contact with water or even at high humidity and their release is associated with an increased outcome of asthma symptoms e.g. during thunderstorms [2]. Because of their size (d < 5 μm) SPP can deposit in the alveolar region of the lung subsequent to inhalation [3]. Since this region is completely covered with the pulmonary surfactant layer which is defined as a surface-active complex of lipids and proteins, deposited particles are directly translocated to the aqueous hypophase due to wetting forces upon impingement [4,5]. Importantly, the hypophase is the compartment of hydrophilic surfactant protein D (SP-D) and it is known that SP-D is able to bind to SPP [6]. SP-D belongs to the family of the collectins (collagen containing lectins) and is built of 12 monomers (43 kDa) each consisting of a N-terminal region, a collagen-like domain, a neck region and a globular head carbohydrate recognition domain (CRD) [7]. Three of these monomers cluster to trimers (~ 130 kDa) and four of these trimers assemble to SP-D, which is a dodecamer (~ 520 kDa) with a crucifix form. In addition, SP-D can oligomerize into larger multimers (> 1 MDa) [8]. Importantly, binding of SP-D to SPP can lead to an increased phagocytosis of the SPP by alveolar macrophages (MO) [6]. In addition, SP-D modulates the interaction of SPP and airway epithelial cells (EC) as shown in a previous study [9]. Besides MO and EC, dendritic cells (DCs) which realize as sentinels and most competent antigen-presenting cell a surveillance network in the pulmonary tissues are among the key players in initiating and maintaining allergic diseases [10]. However, little is known about their uptake potential of SPP and the influence of SP-D on the down-stream immunological reactions. A previous study described, that SP-D reduced the number of SPP-positive dendritic cells (DCs) [11].

Most of the studies, including the above mentioned, which focused on the interaction of SP-D and allergen particles or other pathogens were accomplished in *in vitro*-monocultures which lack important cell-to-cell interactions to resemble the *in vivo *situation. *In *vivo, cells are able to communicate directly and indirectly with each other in order to orchestrate a specific or unspecific immune response [12-15]. To account for this, we studied the uptake of SPP from timothy grass (*Phleum pratense*) as well as the influence of SP-D in cells within an epithelial airway model, consisting of human monocyte-derived macrophages (MDM), ECs, and human monocyte-derived dendritic cells (MDDC) [16-18]. This complex cell culture model warranted maximal interaction between the participating cells as it has been shown upon exposure of the three cell types to 1 μm polystyrene particles [19]. By using laser scanning microscopy (LSM) and advanced computer-controlled analysis, the percentage of cells which participated in SPP-uptake, as well as the absolute number of allergen particles within single cells was investigated. To evaluate the patho-physiological consequence of the SPP-uptake and the modulation by SP-D, autologues CD4^+^-T-cells were isolated from blood of allergic donors, added to the epithelial airway model, and inflammatory mediators were measured after further incubation.

## Methods

### Material

Rat recombinant SP-D (SP-D) was purified by maltose affinity chromatography from the media supernatant of cultured Chinese hamster ovary cells stably transfected with a full-length rat SP-D cDNA clone as described previously [20]. Timothy grass (*Phleum pratense*) pollen were obtained from Allergon (Ängelholm, Sweden). All other reagents, unless otherwise specified, were purchased from Sigma Chemical (Deisenhofen, Germany).

All subjects who donated blood gave their written consent after being fully informed about the purpose and nature of the studies which were approved by the Ethics Committee of Hannover Medical School.

### Subpollen particles

SPP were isolated from timothy grass pollen as described previously [6]. 300 mg of pollen were shaken and vortexed in 40 ml of deionized, autoclaved water for 3 min. Whole pollen and pollen fragments were then separated by centrifugation at 50 *g *for 4 min. The supernatant was filtered (5 μm filter, VWR International, Hannover, Germany) and centrifuged twice at 2500 *g *for 10 min. The resulting pellet was resuspended in 1 ml of sterile NaHCO_3 _(0.1 M) for fluorescence labelling or phosphate buffered saline (PBS) for direct use in the experiments. To determine the number of SPP, an aliquot was diluted in PBS (1:100) and then counted in an *improved Neubauer chamber*.

Immediately after the isolation procedure, SPP were fluorescently labelled with Alexa Fluor 488 fluorescent dye (Molecular Probes, Eugene, OR). For the staining procedure an amount of 1 × 10^9 ^- 2 × 10^9 ^SPP in 1 ml NaHCO_3 _(0.1 M) was used. The suspension was transferred into the vial of reactive dye and rotated for 1 h at room temperature in the dark. Sterile PBS (14 ml) was added, centrifuged at 2500 *g *for 12 min and the pellet was resuspended in 1 ml of PBS. The SPP were counted under fluorescence light in an *improved Neubauer chamber*.

During this study, measurement of Lipopolysaccharide (LPS) contamination of the SPP was not performed.

### A549 culture

A549 cells were obtained from the American Type Culture Collection (ATCC) and cultured in RPMI 1640 Medium (Cambrex Bio Sciences, Walkerswille, MD) supplemented with 10% heat-inactivated foetal calf serum (FCS) and 1% penicillin-streptomycin in a 37°C humidified atmosphere with 5% CO_2_.

### Triple cell co-cultures

**Figure 1:**
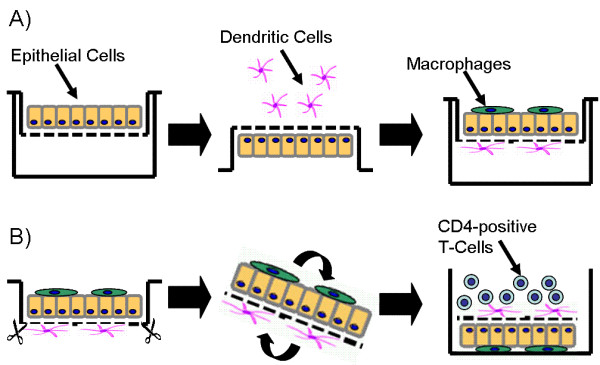
**Epithelial airway model**. A) Creation of the triple cell co-culture model. B) Membrane with the epithelial airway model was excised, turned around and CD4+-T-cells were added.

Cultures were prepared as previously described [16] and as shown in Figure 1A. Briefly, A549 cells (passage 10-40) were grown on cell culture inserts (surface area of 4.2 cm^2^, pores of 3.0 μm in diameter, high pore density PET membranes for 6-well plates; BD Biosciences, Basel, Switzerland). Macrophages and dendritic cells were derived from human blood monocytes as described before [16]. Briefly, peripheral blood monocytes were isolated from buffy coats (blood donation service, Bern, Switzerland) or whole blood (Fraunhofer ITEM, Hannover, Germany) and cultured in the same medium as used for the epithelial cells except for the supplementation of 5% human serum (blood donation service Bern, Switzerland and Invitrogen, Karlsruhe, Germany) instead of 10% foetal calf serum. For the generation of MDDC the monocytes were cultured for 7d in medium supplemented with 34 ng/mL IL-4 (Sigma, FlukaChemie GmbH, Buchs, Switzerland) and 50 ng/mL GM-CSF (R&D Systems, Oxon, UK), whereas MDM were obtained from peripheral blood monocytes cultures without additional supplements for 7 days. A549 epithelial cells were cultured for 7 days before MDM were added on top of the epithelial monolayer and finally MDDC were added underneath the insert membrane. The triple cell co-cultures were kept overnight in medium supplemented with 1% L-Glutamine, 1% penicillin/streptomycin, and 5% heat inactivated (pooled) human serum at 37°C in 5% CO_2 _humidified atmosphere. Triple cell co-cultures were incubated for 8 hours with 10 × 10^6^Alexa488 labelled SPP which were added to the apical chamber for uptake quantification or with unlabelled SPP for subsequent cytokine/chemokine determination.

### Quadruple cell co-cultures

The isolation of immune cells was done from blood of Phleum pratense sensitized atopic humans for in vitro experimentation. Subjects had to have a history of allergy to grass pollen and a positive skin prick test for Phleum pratense pollen at or within 12 months prior to their visit. Autologous CD4^+^-T-cells were isolated according to the manufactures instructions (CD4^+^-T-cell Isolation Kit II, Miltenyi Biotec, Bergisch Gladbach, Germany) and subsequently stored for 1 week at -80°C. After 8 hours incubation of the triple cell co-cultures with unlabelled SPP and SP-D, thorough washing steps with phosphate buffered saline solution (PBS) were performed. The membrane with the triple cell co-cultures was excised, turned around, and placed in a 12-well plate so that the MDDC resided on the upper side of the membrane (Figure 1B). After thawing the autologous T-cells were added to the epithelial airway model (DC-T-cell proportion: 1:10) in 2 ml medium and incubated for 72 hours.

### Cell labelling and fixation

Cells were washed in PBS and fixed for 15 min at room temperature in 3% paraformaldehyde in PBS. Fixed cells were treated with 0.1 M glycine in PBS for 5 minutes and permeabilized in 0.2% Triton X-100 in PBS for 15 min. The cells were incubated with primary and secondary antibodies for 60 min each at room temperature. Preparations were mounted in PBS:glycerol (2:1) containing 170 mg/mL Mowiol 4-88 (Calbiochem, VWR International AG).

Antibodies were diluted in PBS as follows: mouse anti-human CD14 1:20 (Clone UCHM-1, C 7673, Sigma, Deisenhofen, Germany), mouse anti-human CD86 1:20 (Clone HB15e, 36931A, PharMingen, BD Biosciences), goat anti-mouse cyanine 5 1:50 (AP124S, Chemicon, VWR International AG, Life Sciences, Lucerne, Switzerland), and phalloidin-rhodamine 1:100 (R-415, Molecular Probes, Invitrogen AG, Basel, Switzerland).

### Laser scanning microscopy and image restoration

A Zeiss LSM 510 Meta with an inverted Zeiss microscope (Axiovert 200 M, Lasers: HeNe 633 nm, HeNe 543 nm, and Ar 488 nm) was used. Image processing and visualization was done using IMARIS, a 3D multi-channel image processing software for confocal microscopic images (Bitplane AG, Zurich, Switzerland).

### Particle quantification

After image acquisition the total particle number per scan was counted with the particle tracking software Diacount^® ^(Semasopht, Lausanne, Switzerland) as already described for polystyrene particles of different sizes [21,22] and iron-oxide hybrid nanoparticles [23]. For each experimental sample, cells were randomly scanned with the LSM. The particles were counted within individually defined cell types, which were labelled with specific cell surface markers (CD14 for MDM, CD86 for MDDC, and F-Actin for the EC). One observer performed the particle quantification.

### Cytokine/Chemokine determination

Supernatants were taken after 8 hours incubation with unlabelled SPP and further 72 hour incubation with CD4^+^-T-cells in fresh medium. All cytokines and chemokines were measured by bead-based protein quantification. A Bioplex 200 System (Biorad, München, Germany) and a Milliplex bead kit (Millipore, Schwalbach, Germany) were used according to the manufacturer's instructions. Since the values of Interleukin (IL)-8 were above the limit of quantifications, cell culture supernatants were diluted and IL-8 was measured by enzyme linked immunosorbent assay (Duoset, R&D Bioscience, Wiesbaden-Nordenstadt, Germany) according to the manufacturer's instructions. The limit of detection was the lowest standard value of the respective cytokine/chemokine. The limit of quantification was the lowest standard value where the duplicates had a coefficient of variation < 0.2 and a mean which was at least 5 times higher than the blank value.

### Uptake Index

To assess the effect of SP-D, the percentage of positive cells was determined and the number of SPP per cell was counted. In addition, we calculated an uptake index (UI), which considered the percentage of cells containing particles multiplied by the number of SPP inside single cells. The UI was estimated from 6 individual experiments as described by the following formula:

$$\text{UI }=\;\left({\text{C}}_{\left(\text{p}\right)}*{\text{P}}_{\left(\text{c}\right)}\right)$$

C_(p)_: Percentage of cells containing particles. P_(c)_: Number of particles inside cells.

### Statistical analysis

Values are given as means ± SEM. Statistical analysis was performed using GraphPad Prism^®^, Version 4.03. Statistical comparison of the means was performed by ANOVA, followed by a Bonferroni correction. P-values < 0.05 were considered to be significant.

## Results

### Uptake of subpollen particles

**Figure 2:**
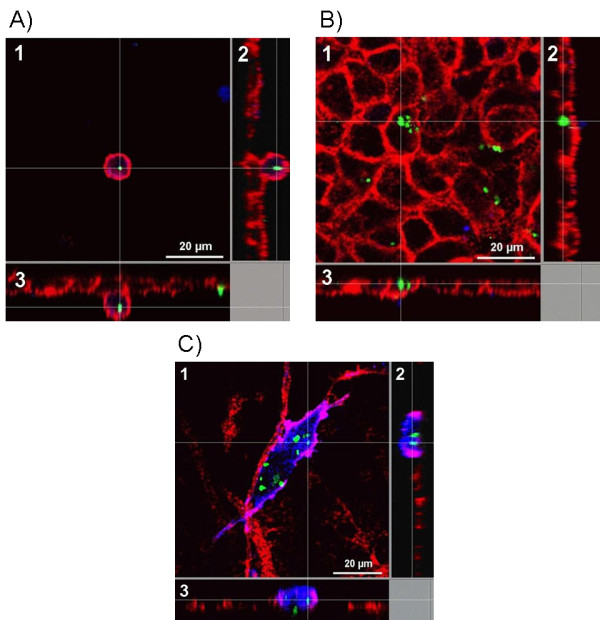
**MDM(A), EC (B), as well as MDDC (C) are able to internalize subpollen particles (SPP)**. In each picture, three dimensions of the cells along the white lines are shown. The intersections display uptaken SPP. The pictures were made by a confocal laser scanning microscope. Green: SPP (Alexa 488); red: F-Actin (Rhodamine phalloidin); blue: CD14 (A), and CD86 (C). 1 represents xy sections, 2 yz and 3 xz.

**Figure 3:**
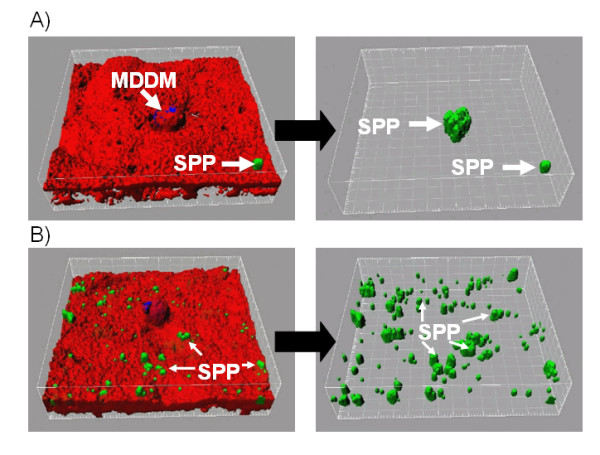
**Pictures displaying the strong modulation of surfactant protein D (SP-D) on distribution of subpollen particles (SPP) within a human epithelial airway model**. Left pictures show an overview of the upper side of the epithelial airway model after incubation with SPP with or without SP-D. Right pictures show internalized and attached SPP after masking of cell borders. A) Epithelial airway model after 8 hours incubation with 10 Million SPPB) Epithelial airway model after 8 hours incubation with 10 Million SPP+ 10 μg/ml SP-D. Green: SPP (Alexa 488); red: F-Actin (Rhodamine phalloidin); blue: CD14. Arrows on the left pictures point to SPP which are attached on the surface of the EC, arrows on the right pictures point to SPP inside cells.

SPP were found intracellulary in MDM (Figure 2A), EC (Figure 2B), and MDDC (Figure 2C), within the human epithelial airway model. Importantly, SP-D was able to modulate this uptake as it can be seen exemplarily in Figure 3.

**Figure 4:**
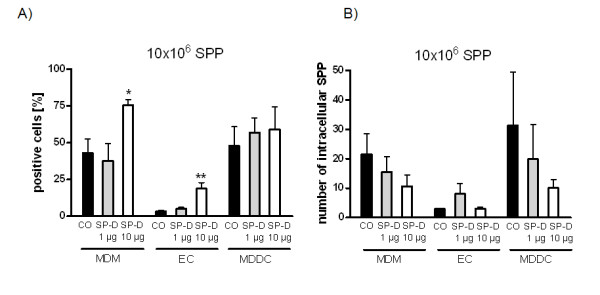
**Influence of surfactant protein D (SP-D) on (A) percentage of cells, within an epithelial airway model, which participated in uptake of subpollen particles (SPP) and (B) on number of intracellular subpollen particles (SPP) in single cells**. Analysis was performed after 8 hours incubation of the epithelial airway model with 10 million SPP by confocal microscopy. CO: Control; MDM: Monocyte derived macrophages; EC: Epithelial Cells; MDDC: Monocyte derived dendritic cells Means of at least 5 experiments ± SEM are shown. * p < 0.05; ** p < 0.01

A detailed quantification revealed that 43.0 ± 9.8% of the MDM took up 21.5 ± 7.2 SPP per cell after 8 hours incubation with 10 million SPP. Furthermore, 3.3 ± 0.7% of the EC took up 2.9 ± 0.3 SPP, and 47.9 ± 13.0% of the MDDC internalized 31.2 ± 18.2 SPP on average (Figure 4). A significantly increased percentage of particle-positive cells was found after co-incubation of the SPP with SP-D (Figure 4A). Whereas 1 μg SP-D/ml did not lead to a significant modulation, 10 μg SP-D/ml increased the percentage of SPP-positive MDDC to 75.4 ± 4.1% (p < 0.05) and the percentage of SPP-positive EC to 18.7 ± 4.1% (p < 0.01). The percentage of MDDC, which were positive for SPP, was not significantly modulated by SP-D.

In contrast, the number of SPP, which were taken up by individual MDM and MDDC, was lower after co-incubation with SP-D compared to control conditions without SP-D (Figure 4B). SP-D (1 and 10 μg/ml) reduced the number of intracellular SPP to 15.5 ± 5.2 and 10.7 ± 3.8 SPP, respectively. The number of SPP in MDDC was decreased to 19.9 ± 11.7 after co-incubation with 1 μg/ml SP-D and to 10.1 ± 2.8 SPP after incubation with 10 μg/ml SP-D. The amount of SPP in EC stayed low and unchanged after co-incubation with SP-D [1-10 μg/ml].

The uptake index (UI) for SPP in MO after incubation with 10 million SPP, i.e. 685.4 ± 193.2, stayed nearly constant after co-incubation with increasing concentrations of SP-D (Table 1). No dose-response was detected. Interestingly, the UI of EC was higher after co-incubation with 1 μg/ml and 10 μg/ml SP-D and increased from 9.9 ± 2.5 to 55.8 ± 32.8 and 64.1 ± 26.4, respectively. However, these increases were not significant. In contrast, the UI of MDDC decreased from 1982 ± 1346 to 1018 ± 455.9 and 570.6 ± 232.2 after co-incubation with SP-D [1 and 10 μg/ml]. Again, these changes were not significant.

**Table 1:** Modulation of Uptake Index by surfactant protein D (SP-D)

Uptake Index	0 μg/ml SP-D	1 μg/ml SP-D	10 μg/ml SP-D
**MDM**	685.4 ± 193.2	511.3 ± 212.7	815.5 ± 289.9

**EC**	9.9 ± 2.5	55.8 ± 32.8	64.1 ± 26.4

**MDDC**	1982 ± 1346	1018 ± 455.9	570.6 ± 232.2

### (Pro-) Inflammatory Response

**Figure 5:**
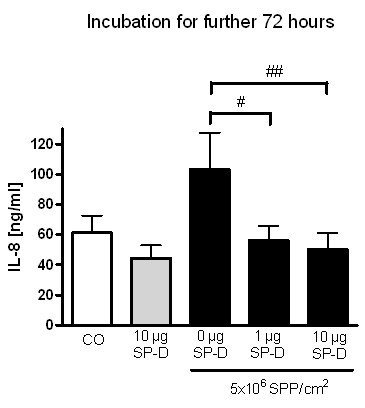
**Secretion of Interleukin (IL)-8 into supernatants of an epithelial airway model after 8 hours incubation with 10 million subpollen particles (SPP) ± surfactant protein D (SP-D) and further 72 h incubation with fresh medium without particles and proteins**. IL-8 was measured by enzyme linked immunosorbent assay. Values are means ± SEM from of least 5 experiments. # p < 0.05; ## p < 0.01; Detection limit was 3.2 pg/ml; Limit of quantification was 64 pg/ml.

Incubation with 10 million SPP for 8 hours increased secretion of IL-8 when cells were incubated for additional 72 hours with fresh medium in the presence of CD4^+^-T-cells. The baseline value of 61.3 ± 11.3 ng/ml, measured in the supernatants of the cells alone, was increased to 103.3 ± 24.3 ng/ml. Importantly, co-incubation with SP-D during the 8 hour-particle exposure period decreased IL-8 secretion significantly (SP-D 1 μg/ml: 56.0 ± 9.8 ng/ml and SP-D 10 μg/ml: 50.5 ± 10.5 ng/ml/Figure 5).

In addition, cells of the epithelial airway model secreted various other cytokines and chemokines (Table 2). After the 72 hours incubation period with fresh medium which followed the 8 hour incubation period with 10 million SPP increased mediator-levels were recorded. SPP exposure significantly increased secretion of IL-1 alpha compared to untreated cells (108.2 ± 22.0 pg/ml versus 170.6 ± 40.6 pg/ml) and Macrophage Inflammatory Protein 1 (MIP-1) beta (1211 ± 105.6 pg/ml versus 1408 ± 143.6 pg/ml). An increase by trend was observed for Granulocyte-Colony Stimulating Factor (G-CSF), Tumor Necrosis Factor (TNF)-alpha, and IL-6. In contrast to IL-8, the secretion of these mediators was not significantly modulated by co-incubation with surfactant protein D [1-10 μg/ml].

**Table 2:** Secretion of cytokines and chemokines after 8 hours incubation with subpollen particles (SPP) plus surfactant protein d (SP-D) and further 72 h incubation with fresh medium in the presence of T-cells

	Cells	10 × 10^6 ^SPP	10 × 10^6 ^SPP + 1 μg/ml SP-D	10 × 10^6 ^SPP +10 μg/ml SP-D	10 μg/ml SP-D
**IL-1 alpha**	108.2 ± 22,0	170.6 ± 40.6*	149.6 ± 33.7	192.7 ± 57.4	102.0 ± 25.6

**G-CSF**	1853 ± 434.3	2823 ± 623.2	2665 ± 566.7	2534 ± 528.2	2596 ± 560.8

**TNF-alpha**	150.9 ± 43.2	339.4 ± 118.5	250.2 ± 102.4	268.8 ± 103.3	235.3 ± 128.6

**MIP-1beta**	1211 ± 105.6	1408 ± 143.6*	1297 ± 233,5	1448 ± 183.4	1224 ± 190.0

**IL-6**	1119 ± 295.9	2327 ± 577.3	1717 ± 284.8	1812 ± 1388.5	1051 ± 151.7

Values are shown as means of at least 8 experiments ± SEM and are given in pg/ml. Detection limit was 3.2 pg/ml. Limit of quantification was: IL-1 alpha 80 pg/ml; G-CSF 80 pg/ml; TNF alpha 80 pg/ml; MIP-1 beta 80 pg/ml; IL-6 16 pg/ml; * p < 0.05 vs Cells

## Discussion

The present study describes for the first time the influence of SP-D on cellular uptake of allergen particles such as SPP within a complex lung cell culture model and the secretion of cytokines/chemokines. Our data show that SP-D increased the number of MDM and EC, which participated in allergen particle uptake. Interestingly, the number of SPP per single cell did not increase upon incubation with increasing SP-D concentration. Using an uptake index (UI) that considered both, the percentage of cells containing particles as well as the number of SPP inside single cells, we demonstrated that the total number of SPP stayed constant in MDM, was increased in EC, and was decreased in MDDC. Incubation with SPP increased secretion of (pro-)inflammatory cytokines and chemokines. SP-D inhibited the IL-8 release from the cells.

Little is known about the uptake of naturally occurring allergen particles by resident lung cells. In addition, although SP-D is one of the first proteins which comes into contact with inhaled particles and thereby modulates immune responses [24], only few data exist describing the effects of a SP-D-particle interaction mainly leading to a increased percentage of alveolar macrophages which took up allergen particles [6,11]. However, the same study also showed that SP-D reduced the number of SPP-positive tissue macrophages and DCs 24 hours after in vivo instillation of the particles [11]. These results highlight the potential differences between effects observed *in vitro *and *in vivo *and further emphasize the need of complex *in vitro *systems which may much better mimic specific *in vivo *situations. In fact, our results show a decreased UI for the MDDC which would be in line with the results of the study of Winkler and co-workers considering a substantial amount of particle clearance during the longer observation period they used [11]. Within the present study, it is important to note, that the measurement of particle quantification was only performed by a single observer. Although the software Diacount^® ^does hardly allow for a subjective counting, this should be taken into consideration. Importantly, we were able to reliably determine SPP uptake in contrast to binding and focused only on the intracellular SPP. Recently we showed that SP-D increased the amount of human primary bronchial epithelial cells with attached SPP [9]. It is further known that the uptake of particle-like surfactant-aggregates in type II pneumocytes is increased upon co-incubation with SP-D [25]. In accordance, we found an increased amount of EC which took up SPP and also an increased UI upon incubation with SP-D. Importantly, in a previous study [9], we found that SP-D was able to modulate the interaction of SPP with human primary bronchial epithelial cells but not with A549 cells. Interestingly, the A549 cells, incorporated in the present epithelial airway model, reacted much more sensitive upon contact with SP-D and SPP compared to the A549 in vitro monocultures [9]. Hence we may speculate that contact to other lung cells such as immune cells modulates the A549 cells to react more similar than human primary epithelial cells.

Incubation with 10 million SPP led to an increased (pro-)inflammatory response within the epithelial airway model after further 72 hours incubation with fresh medium compared to cultures without SPP. This clearly indicates an inflammatory effect of SPP that may also occur in the lung after a single inhalation of allergen particles and that may be independent of allergic sensitization to the particles. The increased secretion of several cytokines (e.g. IL-8) determines a pro-inflammatory action of the SPP which was previously observed for various other allergens in different experimental systems [26-30]. Interestingly, some of these mediators are even released by cells upon contact to inert particles. It is e.g. known, that polystyrene particles induce the secretion of TNF-alpha [31] or IL-8 [32]. Furthermore, SPP are a natural material and are consequently contaminated by LPS. This contamination is of course at least partly responsible for the secretion of inflammatory cytokines. However, it was the aim of the study to investigate the allergen particles as they occur in the nature and not only by means of a purified laboratory situation. Hence we conclude that the secretion of (pro-)inflammatory mediators belongs to all three parameters: the nature of the allergen itself, the particulate body of the allergen particles, and possible LPS contamination.

Importantly, an allogenic response within the co-culture model can be excluded. With respect to IL-8, we observed cytokine secretion after measuring just the cell culture system without any stimulus. However, after incubating the cell-culture system with SPP, we found a significant increase of IL-8. Hence we conclude that this increase is not the result of an allogenic stimulation. The addition of SP-D decreased this SPP-modulated increase of IL-8 which further supports the notion of being no allogenic effect.

SP-D led to a significantly decreased level of IL-8 in the supernatant in the cell cultures after incubation. This is in contrast to a former study with primary bronchial epithelial cells in monocultures. It has been observed that epithelial mono-cultures and triple cell co-cultures react differently upon exposed to various particles e.g. diesel exhaust particles, titanium dioxide as well as single-wall carbon nanotubes [33,34]. We hypothesize that there is a synergistic effect due to the interaction of the three cell types (EC, MDM and MDDC) that reduce the adverse effects of the xenobiotica. It is important to note that different cell types, included in a complex cell culture model, are better at simulating the real situation in the lung than mono-cultures. Hence we believe, that the present results simulate more realistic the in vivo situation. In a former study performed with fixed co-cultures of MDM placed on top and MDDC placed below a monolayer of EC and exposed to 1 μm polystyrene particles, we were able to visualize frequent interactions between these two cell types. MDM and MDDC were found to extend cytoplasmic processes across the epithelial barrier building cell-cell contacts, and particles were found in MDM and MDDC, some of them near cell-cell contacts [35]. Another study revealed the expression of tight junction and adherens junction protein in MDM and MDDC which was suggested to preserve the epithelial integrity in a trans-epithelial network maintained by both immune cells in order to capture and translocate inhaled particulate antigen through the epithelial lung barrier [36]. These ways of communication have to be investigated in more detail in further studies in order to understand cellular interactions in cell culture models.

Importantly, the cell cultures within these experiments were observed under a light microscope after the end of each experiment. Thereby, we could clearly see the normal cell shape with no signs of apoptotic blebbing or disturbance in the confluent A549 cell layer. In addition, our positive control Concanvalin A led to a secretion of most of the measured cytokines and chemokines. From dead cells, the secretion would not be so pronounced.

T-cell-dependent mediators were measured 72 hours after adding of the CD4^+^-T-cells, too (see additional file 1, table S1). Although our positive control (Concancavalin A) induced a release of most of the mediators (see additional file 1, table S1), we were not able to measure a significant release after incubation with SPP. For us, the most likely explanation of the low concentrations is the low proportion of MDDC to allergen (*Phleumpratense*)-specific T-cells. However, it is important to note that up till now we were only able to cultivate the T-cells within the co-culture model for 72 hours in order to guarantee viability of all cells. Crucial parameters of the immunological responses within the body normally occur over longer time periods. This limitation has to be considered. Thereby, the epithelial airway model should be further optimized so that a standardized use of the four different cell types is warranted and T-cell-dependent mediators and behaviour can be evaluated.

## Conclusion

Taken together, SPP uptake in various cell types, i.e. MDM, EC and MDDC, of a human epithelial airway model is modulated by SP-D. Thereby, a SPP-induced inflammation is decreased by SP-D. In addition, the contact to other lung cells modulates the A549 cells to react more similar than primary lung cells.

## List of abbreviations

EC: Epithelial Cell; MDM: Monocyte derived Macrophage; MDDC: Monocyte derived Dendritic Cell; PSG: Pollen Starch Granules; SPP: Subpollen Particle; SP-D: Surfactat Protein-D

## Competing interests

The authors declare that they have no competing interests.

## Authors' contributions

CS planned the concept and study design, performed the experiments, interpreted the results and wrote major parts of the manuscript. BMR planned the concept and study design, interpreted the results and wrote major parts of the manuscript; VJE planned the concept and study design and interpreted the results, PG, FB, HDL, MN, AB, and NK made substantial contributions to the analysis and interpretation of the data. JMH planned the concept and study design, made substantial contributions to the analysis and interpretation of the data and wrote major parts of the manuscript. All of the authors have critically read the manuscript and approved its submission.

## Supplementary Material

Additional file 1 **Table S1**. Secretion of cytokines and chemokines after 8 hours incubation with subpollen particles (SPP) plus surfactant protein d (SP-D) as well as further 72 h incubation with fresh medium. Click here for file
