# Supplementary material for: Surfactant Protein D modulates allergen particle uptake and inflammatory response in a human epithelial airway model
Source: Respir Res. 2012 Feb 1;13(1):8. doi: 10.1186/1465-9921-13-8 (PMC3295667; doi:10.1186/1465-9921-13-8)
Supplement: Additional file 1 — Table S1. Secretion of cytokines and chemokines after 8 hours incubation with subpollen particles (SPP) plus surfactant protein d (SP-D) as well as further 72 h incubation with fresh medium. [file 1465-9921-13-8-S1.DOC]

**Supplementary File**

Supplementary Table S1: Secretion of cytokines and chemokines after 8 hours incubation with subpollen particles (SPP) plus surfactant protein d (SP-D) as well as further 72 h incubation with fresh medium.

|  | **Control** | **10x10^6^ SPP** | **10x10^6^ SPP + 1 µg/ml SP-D** | **10x10^6^ SPP +**  **10 µg/ml SP-D** | **10 µg/ml**  **SP-D** | **Con A (positive control)** |
| --- | --- | --- | --- | --- | --- | --- |
| **IL-2** | b.q.l. | b.q.l. | b.q.l. | b.q.l. | b.q.l | 1530 ± 248 |
| **IL-4** | b.q.l. | b.q.l. | b.q.l. | b.q.l. | b.q.l | 607 ± 552 |
| **IL-5** | n.d. | n.d. | n.d. | n.d. | n.d. | 166 ± 56 |
| **IL-10** | b.q.l. | 28.6 ± 12.1 | 24.1 ± 11.8 | 17.5 ± 5.8 | 23.8 ± 15.7 | 344 ± 214 |
| **IL-12p70** | n.d. | b.q.l. | b.q.l. | b.q.l. | n.d. | 43 ± 18 |
| **IL-13** | b.q.l. | b.q.l. | b.q.l. | b.q.l. | b.q.l | 976 ± 255 |
| **IFN-gamma** | b.q.l. | 95,0 ± 69.2 | b.q.l. | b.q.l. | b.q.l | 2699 ± 1010 |
| **IP-10** | 476.8 ± 247.3 | 774.1 ± 265.4 | b.q.l. | 676.6 ± 245.7 | 620.0 ± 215.1 | 998 ± 266 |

Values are shown as means of at least 8 experiments ± SEM and are given in pg/ml. Detection limit was 3.2 pg/ml. Limit of quantification was: IL-2 16 pg/ml; IL-4 80 pg/ml; IL-5 3.2 pg/ml; IL-10 16 pg/ml; IL12p70 80 pg/ml; IL-13 16 pg/ml; IFN-gamma 80 pg/ml; IP-10 400 pg/ml; b.q.l.: below quantification limit; n.d.: not detected; Con A: Concanvalin A (Con A was given during the 72h incubation period with fresh medium).
